# Spatial and Temporal Evolutionary Patterns in Puumala Orthohantavirus (PUUV) S Segment

**DOI:** 10.3390/pathogens9070548

**Published:** 2020-07-08

**Authors:** Florian Binder, René Ryll, Stephan Drewes, Sandra Jagdmann, Daniela Reil, Melanie Hiltbrunner, Ulrike M. Rosenfeld, Christian Imholt, Jens Jacob, Gerald Heckel, Rainer G. Ulrich

**Affiliations:** 1 Friedrich-Loeffler-Institut, Federal Research Institute for Animal Health, Institute of Novel and Emerging Infectious Diseases, 17493 Greifswald-Insel Riems, Germany; binderflorian@aol.com (F.B.); ryllrene@web.de (R.R.); stephan.drewes@fli.de (S.D.); sandra.jagdmann@web.de (S.J.); ulrike.rosenfeld@gmx.de (U.M.R.); 2 Cheplapharm Arzneimittel-GmbH, 17489 Greifswald, Germany; 3 Institute for Medical Immunology, Charité-Universitätsmedizin Berlin, Corporate member of Freie Universität Berlin, Humboldt-Universität zu Berlin, Berlin Institute of Health, 13353 Berlin, Germany; 4 Julius Kühn-Institut, Federal Research Centre for Cultivated Plants, Institute for Plant Protection in Horticulture and Forests, Vertebrate Research, 48161 Münster, Germany; daniela.reil@uni-potsdam.de (D.R.); christian.imholt@julius-kuehn.de (C.I.); jens.jacob@julius-kuehn.de (J.J.); 5 Institute of Ecology and Evolution, University of Bern, 3012 Bern, Switzerland; melanie.hiltbrunner@iee.unibe.ch (M.H.); gerald.heckel@iee.unibe.ch (G.H.)

**Keywords:** hantavirus, bank vole, evolution, N protein, NSs protein, S segment

## Abstract

The S segment of bank vole (*Clethrionomys glareolus*)-associated Puumala orthohantavirus (PUUV) contains two overlapping open reading frames coding for the nucleocapsid (N) and a non-structural (NSs) protein. To identify the influence of bank vole population dynamics on PUUV S segment sequence evolution and test for spillover infections in sympatric rodent species, during 2010–2014, 883 bank voles, 357 yellow-necked mice (*Apodemus flavicollis*), 62 wood mice (*A. sylvaticus*), 149 common voles (*Microtus arvalis*) and 8 field voles (*M. agrestis*) were collected in Baden-Wuerttemberg and North Rhine-Westphalia, Germany. In total, 27.9% and 22.3% of bank voles were positive for PUUV-reactive antibodies and PUUV-specific RNA, respectively. One of eight field voles was PUUV RNA-positive, indicating a spillover infection, but none of the other species showed evidence of PUUV infection. Phylogenetic and isolation-by-distance analyses demonstrated a spatial clustering of PUUV S segment sequences. In the hantavirus outbreak years 2010 and 2012, PUUV RNA prevalence was higher in our study regions compared to non-outbreak years 2011, 2013 and 2014. NSs amino acid and nucleotide sequence types showed temporal and/or local variation, whereas the N protein was highly conserved in the NSs overlapping region and, to a lower rate, in the N alone coding part.

## 1. Introduction

The family *Hantaviridae*, order *Bunyavirales*, comprises rodent, insectivore and bat-borne viruses. The enveloped virion of about 80–120 nm in diameter contains three genome segments of single-stranded RNA with negative polarity [1]. The large (L) segment encodes the RNA-dependent RNA polymerase (RdRP). The medium (M) segment encodes the glycoprotein precursor that is co-translationally processed and cleaved into the two glycoproteins, Gn and Gc. The small (S) segment encodes the nucleocapsid (N) protein, which is the most abundant hantaviral protein. The N protein is involved in multiple processes during the replication cycle and is highly immunogenic and therefore, is used as an antigen in serodiagnostics [2]. In addition, the S segment of Puumala orthohantavirus (PUUV) and other vole-associated hantaviruses encodes, in a second, overlapping open reading frame (ORF), a non-structural (NSs) protein. This NSs protein was shown to be functional and involved in suppression of interferon production and related mechanisms for antiviral activity [3]. Recent studies in Lower Saxony, Germany, confirmed the conservation of a putative NSs-ORF in PUUV strains concerning its position within the S segment and length of 270 nucleotides [4,5].

PUUV is the geographically most widely distributed human pathogenic hantavirus in Europe. It is the main causative agent of hemorrhagic fever with renal syndrome (HFRS) and its milder form, nephropathia epidemica (NE). The natural host of PUUV is the bank vole (*Clethrionomys glareolus* syn. *Myodes glareolus*) that is commonly believed to be persistently infected without signs of disease [6]. However, one investigation of bank vole populations on Finnish islands indicated a slightly decreased overwinter survival rate of PUUV-infected bank voles compared to non-infected animals [7]. Bank voles are distributed across large areas of Eurasia and infections with PUUV are recorded in almost all parts of the continent [8,9]. Transmission to humans occurs by inhaling aerosols comprised of dried saliva, feces and urine of virus-infected rodents. Furthermore, a rare transmission route for the virus can be rodent bites [10]. Bank vole abundance in Central Europe was shown to fluctuate depending on beech mast intensity in the previous year [11]. The proportion of PUUV infections within host populations is influenced by the population density of bank voles, habitat properties and the presence of maternal antibodies in juvenile voles [12,13,14].

In Europe, PUUV disease outbreak years are characterized by a high incidence of human PUUV cases and occur roughly every 2–3 years depending on host dynamics. In Germany, increased numbers of notified cases were observed in the outbreak years 2007 (1678 cases), 2010 (2016), 2012 (2852), 2017 (1697), and 2019 (1530) [9,15]. The occurrence of PUUV in Germany is tied to the occurrence of the Western evolutionary lineage of the bank vole [16,17], which is present in Southern and Western Germany and absent in the eastern part of Germany. Phylogenetic analysis typically shows geographical clustering of PUUV S segment sequences independent of the year of collection [18,19]. Analysis of the temporal variation of PUUV sequences in the bank vole population demonstrated the continuous presence of certain sequence types over multiple years, whereas other minor sequence types seemed to emerge and disappear again [20]. 

**Figure 1:**
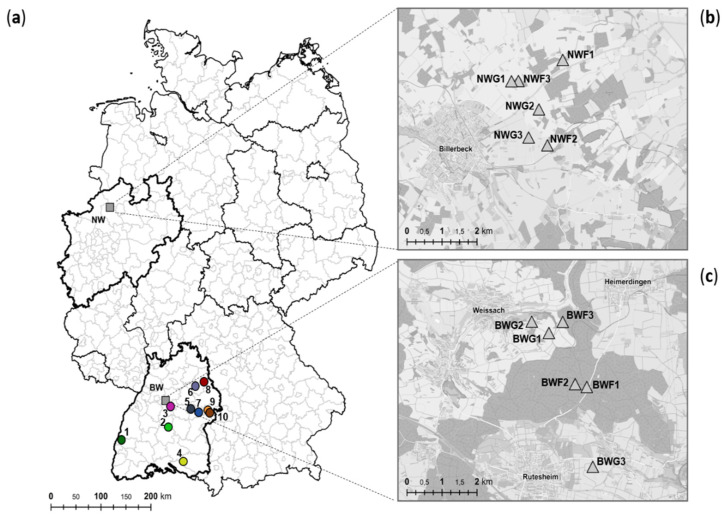
Location of study sites for Puumala orthohantavirus (PUUV) analysis in Germany (**a**) with trapping plots in North Rhine-Westphalia (NW-mo) (**b**) and Baden-Wuerttemberg (BW-mo) (**c**). Study sites consisted of six trapping plots, three in forest (F) and three in grassland (G) habitats. Single plots of trapping regions in BW and NW are shown as triangles. (**b**) NW-Billerbeck: NWF1 (51.998752 North (N) latitude, 7.335144 East (E) longitude), NWF2 (51.978863 N, 7.329545 E), NWF3 (51.993606 N, 7.316983 E), NWG1 (51.993511 N, 7.314114 E), NWG2 (51.987140 N, 7.325719 E), NWG3 (51.980575 N, 7.321710 E). (**c**) BW-Weissach: BWF1 (48.829307 N, 8.966542 E), BWF2 (48.829981 N, 8.962033 E), BWF3 (48.844419 N, 8.957213 E), BWG1 (48.841850 N, 8.951865 E), BWG2 (48.844470 N, 8.945196 E), BWG3 (48.810801 N, 8.968887 E). Additional trapping plots in BW from 2007 and 2012 are shown in (**a**) as circles. 1: Kenzingen (year 2012, 48.11369, 7.50524); 2: Mössingen-Belsen (2012, 48.23227 N, 9.03068 E); 3: Stuttgart-Büsnau (2012, 48.44273 N, 9.03288 E); 4: Zußdorf-Wilhelmsdorf (2007, 47.54104 N, 9.23288 E); 5: Göppingen (2012, 48.724673 N, 9.710510 E); 6: Michelbach (2007, 49.04597 N, 9.46563 E); 7: Geislingen-Stötten (2012, 48.38513 N, 9.52201 E); 8: Crailsheim-Roßfeld (2012, 49.07519 N, 9.59161 E); 9: Steinheim (2012, 48.40546 N, 10.02022 E); 10: Steinheim (2007, 48.6982 N, 10.06625 E). The color code was also used in the phylogenetic tree in Figure 3a.

A previous study in Baden-Wuerttemberg (BW) and North Rhine-Westphalia (NW), Germany, revealed, based on live trapping, that fluctuation of PUUV seroprevalence is dependent on multi-annual dynamics of rodent host abundance [13]. The aim of this parallel snap trapping study in these two endemic regions (Figure 1) was to evaluate the influence of rodent population fluctuation on PUUV prevalence, molecular evolution and genetic diversity of two coding regions of the PUUV S segment in its natural host and the frequency of potential PUUV spillover infections into non-reservoir species.

## 2. Results

### 2.1. Temporal Fluctuation of PUUV Prevalence in Bank Voles from Baden-Wuerttemberg and North Rhine-Westphalia

**Figure 2:**
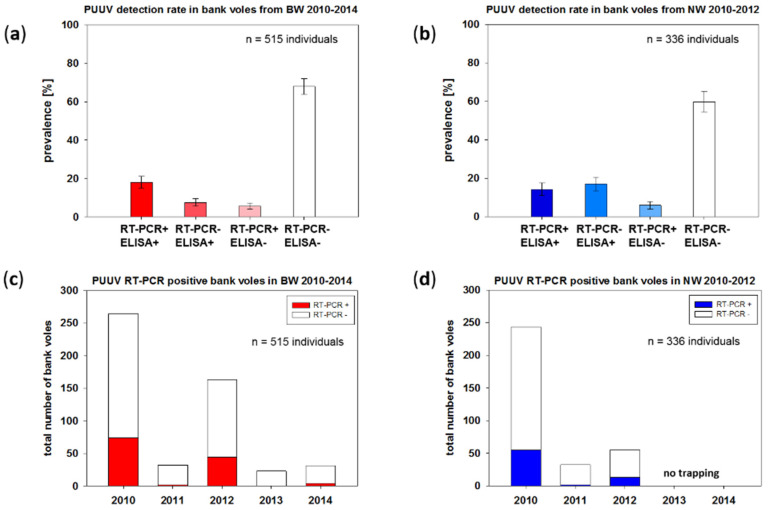
Serology and reverse transcription-polymerase chain reaction (RT-PCR) screening of voles from Baden-Wuerttemberg (**a**) and North Rhine-Westphalia (**b**) and distribution of RT-PCR-positive bank voles over time in Baden-Wuerttemberg (**c**) and North Rhine-Westphalia (**d**). In a and b, results are given in percentage of all voles investigated from the respective trapping region. Animals for which no lung or chest cavity lavage sample could be obtained are left out. RT-PCR- and enzyme-linked immunosorbent assay (ELISA)-positive animals are shown as dark columns, RT-PCR- and ELISA-negative animals are shown as white columns. Only PUUV RNA- or anti-PUUV-positive animals are shown in blue/red scale. In c and d, the total number of bank voles is shown in columns on the y-axis. Chest cavity lavage samples were used for serological testing by immunoglobulin G (IgG) ELISA. For detection of PUUV RNA, a small piece of lung tissue was used for RNA extraction with Qiazol lysis reagent and RT-PCR screening with subsequent sequencing of amplification products.

Results of snap trapping displayed multiannual dynamics similar to parallel live-trapping, as presented in Reil et al. [13]. Reverse transcription-polymerase chain reaction (RT-PCR) screening of all 851 bank vole lung samples and subsequent sequencing of the amplification products resulted in 193 PUUV RNA-positive samples (22.7%, 95% confidence interval (CI) 20.0–25.6%). In none of the bank voles was Tula orthohantavirus (TULV) RNA detected. PUUV-reactive antibodies were detected in 242/851 (28.4%, CI, 25.5–31.6%) of the bank voles (Figure 2). At the plots in BW, 18.1% (CI, 15–21.6%), and at the plots in NW, 14.3% (CI, 11.0–18.4%) of the tested bank voles were positive in both assays, indicating a persistent infection. A total of 5.6% (CI, 4.0–8.0%) in BW and 6.0% (CI, 3.9%–9.0%) in NW showed PUUV RNA but not an anti-PUUV antibody response, indicating an acute infection (Figure 2a). In 39 of 515 (7.6%; CI, 5.6–10.2%) of the bank voles in BW, PUUV-reactive antibodies were detected, but no PUUV RNA (Figure 2a). Four of these seropositive animals were juvenile (≤15 g), which might indicate a presence of maternal antibodies. The other 35 seropositive voles were adults; therefore, the lack of RNA detection might suggest a clearance of the virus from the voles. The investigations of the bank voles from NW indicated a similar picture, although an even larger part, 57 of 336 (17.0%; CI, 13.3–21.4%) of the bank voles, were anti-PUUV antibody-positive, but RT-PCR-negative (Figure 2b). Here, 12 of the 57 seroreactive animals were juveniles. Overall, PUUV RNA detection rates were higher in bank voles during the outbreak years 2010 and 2012 and lower in non-outbreak years 2011, 2013 and 2014 (Figure 2c,d)

### 2.2. Phylogenetic and Isolation-by-Distance Analysis of Non-NSs-Overlapping S Segment Sequences

**Figure 3:**
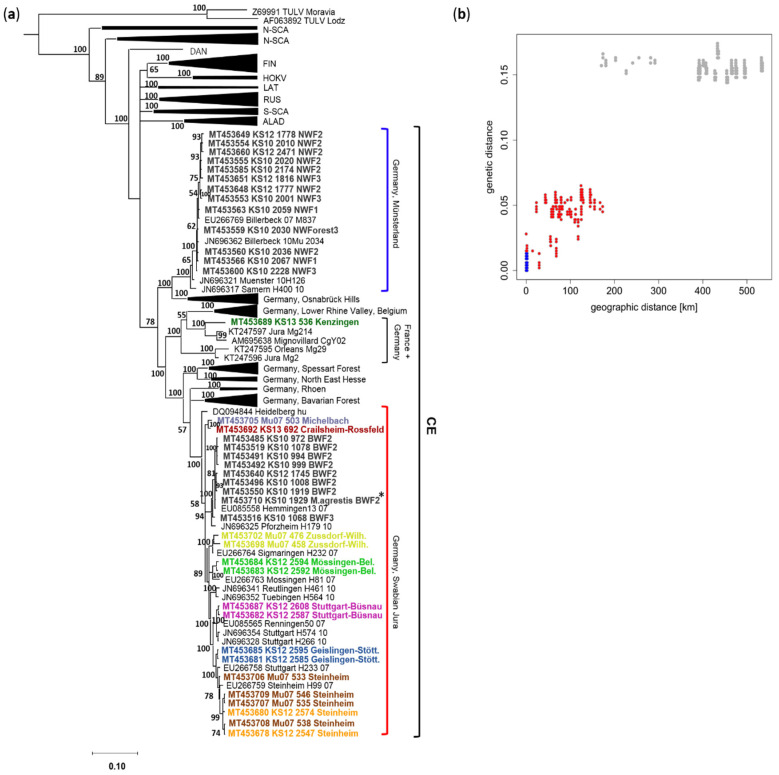
Phylogenetic tree of PUUV strains (**a**) and isolation-by-distance analysis (**b**). The phylogenetic tree (**a**) was constructed on the basis of 213 unique partial sequences of the N protein-encoding but not NSs overlapping S segment region (465 nucleotides, nucleotides 436–900 of the S-segment, numbering according to PUUV strain Sotkamo, accession number HE801633.1). In total, 226 new individual PUUV S segment sequences were generated (see Supplementary Tables S1 and S2) and 39 unique sequences were used for phylogenetic analysis. Analysis was performed using the CIPRES gateway and MrBayes v.3.2.7 [21]. A mixed nucleotide substitution matrix was specified in 4 independent runs of 10^7^ generations. Phylogenetic relations are shown as a maximum clade credibility phylogenetic tree with posterior probabilities for major nodes. Tula orthohantavirus (TULV) sequences Lodz and Moravia were used as outgroup. Sample numbers for identification consisted of the indicated dissection year, section number and trapping site. Novel sequences from this study are indicated by bold font. * = sequence from the spillover infected field vole. For clarity, previously characterized PUUV clades are shown in simplified form [17,18]. NW, North Rhine-Westphalia; BW, Baden-Wuerttemberg; HOKV, Hokkaido; LAT, Latvian; ALAD, Alpe-Adrian; S-SCA, South Scandinavian; N-SCA, North Scandinavian; RUS, Russian; FIN, Finnish; DAN, Danish. The extent of local transmission and evolution of PUUV was investigated by isolation-by-distance patterns in BW and NW (**b**). Pairwise genetic distances were estimated based on the same 465 nucleotide-long S segment region, assuming uniform mutation rates among sites. Statistical significance of the association between the half-matrices of both distance types was estimated with a Mantel test. Coordinates given in Figure 1 were used for isolation-by-distance analysis. Blue dots indicate comparisons between sequences from NW, red dots comparisons between sequences from BW. Grey dots indicate comparisons between the populations in BW and NW that do not show isolation-by-distance patterns due to mutational saturation of sequences [19]. Genetic distance is given as percentage (%), geographic distance in kilometers (km).

For all PUUV RNA-positive bank voles, partial S segment sequences of 1007 nucleotides were generated, covering a major part of the coding sequence of the N protein, including the NSs-overlapping region. Phylogenetic analysis of a 465 nucleotide-long, non-NSs overlapping part of the S segment sequences showed two distinct clades for BW and NW within the Central European (CE) PUUV clade. The sequences from NW clustered together with a previously determined bank vole sequence from the same region from 2007 and sequences from human patients, in sister relationship to the subclade Osnabrück hills, north-west Germany (Figure 3a). The sequences from the BW region clustered in another clade with further bank vole- and human patient-derived sequences from BW, with a close relationship to clades from Rhoen mountains and Bavarian forest (Figure 3a). PUUV sequences from 31 additional animals from nine plots in BW (sites 2–10 in Figure 1a) from the outbreak years 2007 and 2012 (Supplementary Table S1) also clustered in the Swabian Jura clade as the continuously found BWF2/BWF3 sequences. One bank vole-derived sequence from Kenzingen (site 1 in Figure 1a) clustered with sequences from France (Figure 3a).

Genetic differences between PUUV sequences showed a strong positive relationship with geographical distance between sampling locations, resulting in an isolation-by-distance pattern (R² = 0.91; *p* < 0.001; Figure 3b). PUUV S segment sequences from the same phylogenetic clade were up to 7% different within the same sampling region but differed by 14% to 17% between clades or sampling regions. The low number of sampling locations (N = 3) in NW (blue dots) prevented statistical testing for isolation-by-distance in this region, but PUUV sequences from BW (red dots) showed a significant increase of genetic distance between sampling locations less than 120 km apart (R² = 0.596; *p* < 0.001; Figure 3b).

### 2.3. Frequency of Spillover Infections

To evaluate if bank vole population dynamics influence the frequency of PUUV spillover infection, 397 yellow-necked mice (*Apodemus flavicollis*) and 68 wood mice (*A. sylvaticus*) were screened for the presence of PUUV-reactive antibodies and lung tissue samples of 176 common voles (*Microtus arvalis*) and 8 field voles (*M. agrestis*) were investigated by S segment RT-PCR (Supplementary Table S1). Common and field voles were not screened for antibodies as they represent reservoirs of TULV, which cannot be distinguished by immunoglobulin G (IgG) enzyme-linked immunosorbent assay (ELISA) from PUUV. Yellow-necked mice and wood mice were tested for antibodies only, as they were not expected to allow PUUV RNA replication. RT-PCR and subsequent sequencing detected PUUV RNA in one field vole from the outbreak year 2010, but in none of the common voles (Supplementary Table S2). There was high similarity of the field vole-derived sequence to sequences from the same forest plot within the BW clade (BWF2*, Figure 3a). None of the yellow-necked mice and wood mice were found to have PUUV-reactive antibodies (Supplementary Table S2).

### 2.4. Sequence Variability in the N/NSs Overlapping and N Encoding Regions

For all PUUV RNA-positive bank voles, the NSs ORF of 270 nucleotides (90 amino acid residues) was detected in a +1 reading frame relative to the N-encoding ORF. The positions of the start codon and the stop codon as well as the presence and location of multiple translation initiation sites are conserved (Supplementary Figure S1). The stop codon UAA was found for all NSs sequences from NW and site 1 (Kenzingen) in BW, whereas the NSs sequences from all other BW sites have a UGA stop codon.

**Figure 4:**
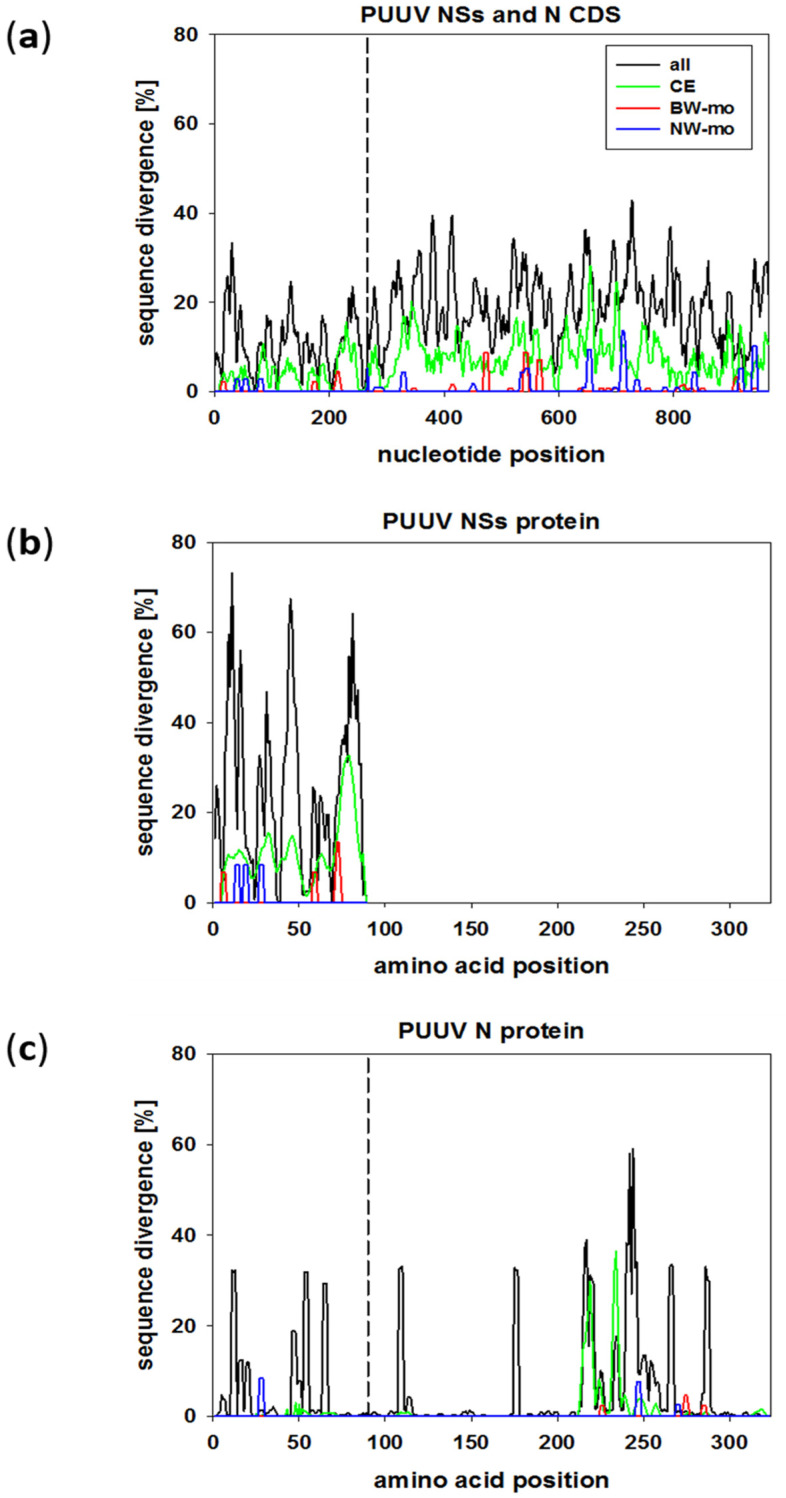
PUUV S segment sequence divergence investigated by SimPlot analysis of the PUUV NSs and N segment nucleotide sequence (**a**), PUUV NSs amino acid sequence (**b**), and PUUV N amino acid sequence (**c**). Sequences from BW (5 unique sequences) are shown in red, sequences from NW (4) in blue, combined sequences from CE clade (35) are shown in green, and an overall PUUV N or NSs SimPlot of 94 sequences from this study and published sequences from all clades from Europe and Asia (see Section 4.4 and details in the text above) is shown as a black line. The N/NSs overlapping region is indicated by a dotted line in (**a**) and (**c**). Divergence is given as a percentage between the compared sequences. Identical sequences are shown in Supplementary Tables S3 and S4. CDS = coding sequence.

Comparison of the NSs and N segment sequences (nt 58–1065) was done group-wise by SimPlot analysis on the nucleotide (Figure 4a) and amino acid level (Figure 4b,c). The NSs sequences of the monitoring trapping regions in BW (BW-mo) and NW (NW-mo) show only little sequence diversity among each other (Figure 4a). To gain insights into the NSs sequence variation in Germany, NSs nucleotide and amino acid sequences from BW-mo and NW-mo trapping sites were compared to additional NSs sequences from other trapping sites in BW and the CE clade (Figure 4, CE). All these sequences from Germany had a low nucleotide sequence divergence in the NSs protein-coding sequence (CDS, Figure 4a, green line). When we compared sequences from all over Europe and Asia (black line), including clades CE, FIN (Finnish), LAT (Latvian), RUS (Russian), DAN (Danish), N-SCA (North Scandinavian), S-SCA (South Scandinavian), ALAD (Alpe-Adrian) and HOKV (Hokkaido), the NSs CDS showed a sequence divergence of 35% at the 5’ end and almost homogeneously distributed peaks of around 20% divergence. This indicates that the NSs CDS is variable among European and Asian PUUV strains, but regional sequences are less divergent. On the amino acid level, the overall pattern was the same, however, variability was particularly high (70%) at NSs amino acid positions 5–15, 40–50, and the C-terminal part of residues 70–90 among all PUUV sequences (Figure 4b). 

The N protein coding ORF was analyzed the same way (Figure 4a,c) and showed largely homogeneously distributed nucleotide sequence divergence. BW-mo and NW-mo sequences each were highly similar, but the CE clade sequences showed peaks of up to 30% nucleotide sequence divergence (Figure 4a) and 40% amino acid sequence divergence (Figure 4c). The NSs overlapping region was more conserved than the N alone coding part of the S segment (Figure 4a,c, compare the left side of dotted line to the right side). The overall average nucleotide divergence for the N/NSs overlapping CDS was 3.9% for the CE clade and 11% for all sequences, whereas the N alone coding part was less conserved, with 6.9% and 19% sequence divergence, respectively (Figure 4a). When translated, PUUV N protein showed high amino acid sequence divergence independent of the NSs overlapping region only at a few regions (Figure 4c). PUUV N protein sequences had a highly variable region at amino acid positions 220–240. However, the main part of the N protein was highly conserved on amino acid sequence level.

### 2.5. Spatial and Temporal Evolution of Different Regions of the PUUV S Segment

In addition to the phylogenetic analysis of partial sequences (nucleotides 436–900), larger S segment sequences spanning nucleotides 58–1065 from lung samples of all 193 PUUV RNA-positive bank voles were analyzed. For this purpose, nucleotide and amino acid sequence types were defined for the NSs + 1 ORF (nucleotides 83–356/complete NSs amino acid residues 1–90, reference PUUV Sotkamo HE801633.1), the overlapping N/NSs coding region (nucleotides 82–355/N protein amino acid residues 13–103) and the N ORF alone (nucleotides 359–1065/N amino acid residues 104–340). Each sequence type was defined as a unique sequence found in a bank vole (or the single field vole) with at least one nucleotide/amino acid residue difference to any other sequence (for details see Supplementary Tables S3 and S4 and Supplementary Figures S1 and S2).

**Figure 5:**
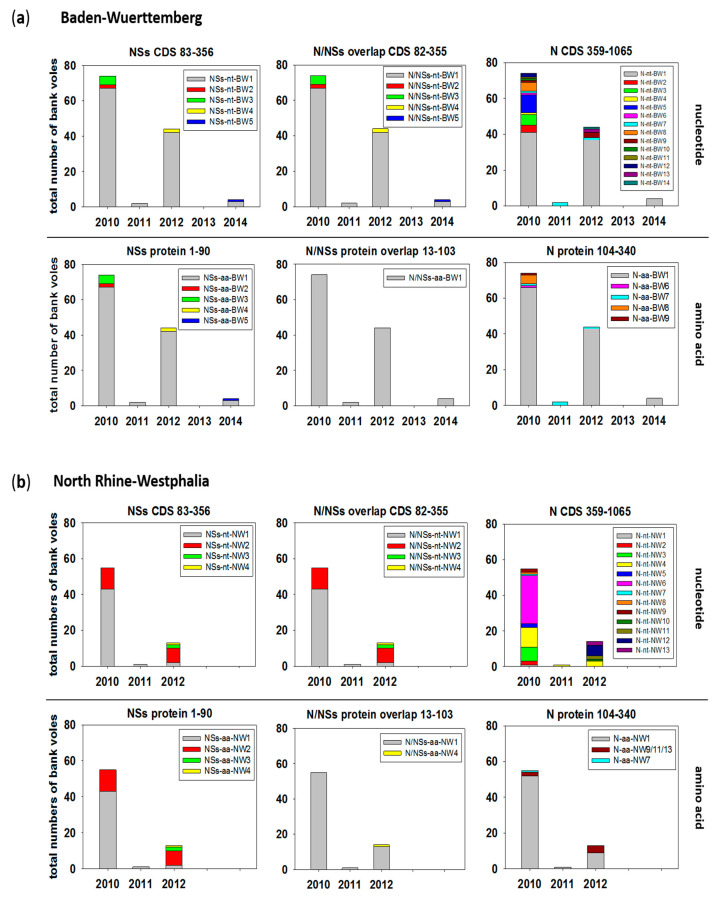
Temporal distribution of PUUV N and NSs sequence types from bank voles collected during 2010–2014 in Baden-Wuerttemberg (**a**) and North Rhine-Westphalia (**b**). The distribution over the trapping period is shown as numbers of bank voles carrying the respective sequence type. A sequence type is defined as a unique sequence found in a bank vole (or field vole) with at least one nucleotide/amino acid difference to any other sequence. Numbers in titles indicate the nucleotide/amino acid positions for the respective ORF analyzed in each panel. Assignment of single sequences to a sequence type is given in Supplementary Tables S3 and S4 and is illustrated for amino acid sequence types in Supplementary Figures S1 and S2. CDS = coding sequence.

In total, the sequences of the NSs coding region and the N/NSs overlapping part from BW represented five types (Figure 5a, NSs-nt-BW1-5; N/NSs-nt-BW1-5), which differed only by one single nucleotide exchange each. Interestingly, when translated, all four single nucleotide exchanges were synonymous for the N/NSs overlap but remained non-synonymous for the NSs-ORF (Figure 5a, N/NSs-aa-BW1, NSs-aa-BW1-5). Similar observations were made for PUUV sequences from the NW region, where the NSs showed three non-synonymous single nucleotide exchanges, resulting in four amino acid sequence types. The N/NSs overlapping nucleotide sequence types (N/NSs-nt-NW1-4) represented a major amino acid sequence type (N/NSs-aa-NW1) and one animal with a single amino acid exchange (Figure 5b, N/NSs-aa-NW4). 

The partial N protein-encoding nucleotide sequence, downstream of the overlapping region (nucleotides 359 to 1065), showed a high number of nucleotide exchanges that resulted in 14 different nucleotide sequence types in BW (Figure 5a) and 13 in NW (Figure 5b). However, nucleotide exchanges were mainly synonymous, resulting in one major amino acid sequence type (N-aa-BW1, N-aa-NW1) and 4 (BW) or 2 (NW) minor types (Figure 5a,b). 

The major sequence type (nucleotide and amino acid) for the NSs alone and N/NSs overlapping coding regions in BW was present over the whole trapping period from 2010–2014 (Figure 5a, NSs-nt/aa-BW1, N/NSs-nt/aa-BW1). Minor sequence types occurred only sporadically in a single year. The N alone coding part (359–1065) also showed a major nucleotide sequence type. However, it was not continuously observed, as in 2011, only two PUUV-infected voles were found representing the minor type (Figure 5a, N-nt-BW7). The same minor type was also present in 2010 and 2012, whereas all other minor types were single occurrences in the outbreak years 2010 and 2012. These nucleotide exchanges were mostly synonymous. However, four minor types were present in outbreak years, including N-aa-BW7 that was found consistently three years in a row (Figure 5a).

Similar to BW, the major NSs and N/NSs sequence types were observed over the whole period in NW (Figure 5b, N-nt/aa-NW1, N/NSs-nt/aa-NW1). Here, the second, more prominent minor type (NSs-nt-NW2) was observed in the outbreak years 2010 and 2012 but disappeared in 2011 (Figure 5b). Concerning the N part, a more diverse picture was observed in NW. On the nucleotide level, one sequence type was observed in all three years (N-nt-NW4, 2010–2012), but this sequence type did not represent a major type, and strains were more equally distributed to several nucleotide sequence types than in BW. Most of these sequence variations were synonymous, except for three minor nucleotide sequence types that had the same amino acid sequence, present in the outbreak years 2010 and 2012 (Figure 5b, N-aa-NW9/11/13). One animal in 2010 showed a single amino acid exchange compared to all others (Figure 5b, N-aa-NW7).

**Figure 6:**
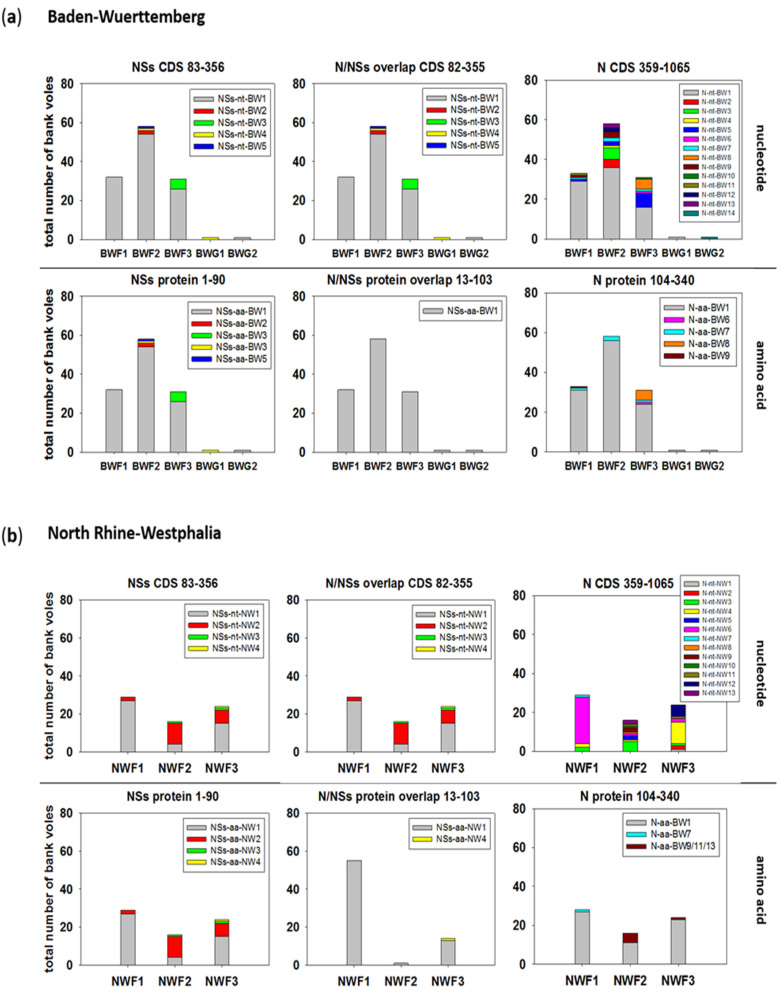
Spatial distribution of PUUV N and NSs sequence types from bank voles collected during 2010–2014 in Baden-Wuerttemberg (**a**) and North Rhine-Westphalia (**b**). The distribution over the trapping plots is shown as numbers of bank voles carrying the respective sequence type. A sequence type is defined as a unique sequence found in a bank vole (or field vole) with at least one nucleotide/amino acid difference to any other sequence. Numbers in titles indicate the nucleotide/amino acid positions for the respective ORF/protein analyzed in each panel. Assignment of single sequences to a sequence type is given in Supplementary Tables S3 and S4 and is illustrated for amino acid sequence types in Supplementary Figures S1 and S2. CDS = coding sequence.

The spatial analysis of NSs sequence types also indicated the occurrence of major nucleotide and amino acid sequence types at all forest plots in BW (Figure 6a). The two PUUV RNA-positive bank voles trapped in grassland habitats carried the main NSs sequence type and a second minor one. At each of the three plots in NW, multiple, mostly unique nucleotide sequence types were observed for the non-NSs overlapping region (N 359–1065), indicating less exchange of bank vole populations between the plots in NW. In the N/NSs overlapping region, two main N/NSs nucleotide sequence types were present at all three forest plots (Figure 6b). Again, the N protein amino acid sequence types were represented by a major type at all forest plots in BW and NW. In contrast, for the NW plots, two main NSs amino acid sequence types were observed, whereas at the forest plots in BW, there was only one major type.

## 3. Discussion

Here, we investigated the influence of fluctuations in bank vole populations on PUUV prevalence, the sequence evolution of different regions of the S segment of PUUV and the frequency of spillover infections in sympatric rodents of other species. 

The trapping regime reflected the multiannual fluctuations with abundance peaks in 2010 and 2012 and low abundance in 2011, 2013 and 2014, similar to the results presented by Reil et al. [13].

The PUUV-RNA detection rate at the site in BW reached 28%, 6.2%, 27%, 0% and 12.9% during 2010–2014, and at the site in NW, 22.6%, 3.1% and 23.6% during 2010–2012. The detection rates during outbreak years 2010 and 2012 are similar to the results of a study in the Bavarian forest during 2004 (RNA detection rate of 24%, [22]), but much lower than those observed during outbreak years at a site in Western Thuringia (77.3%; [23]) and two sites in district Osnabrück, Lower Saxony. Here, the RNA detection rate varied during 2010–2012 between 85%, 13.7% and 86.2% at one site and 100%, 40% and 86.7% at a second site [20]. A preliminary analysis of the seasonality suggests that the ratio of PUUV RNA-positive bank voles was the highest in spring, which mimics the results of the PUUV seroprevalence in live trapping shown by Reil et al. [13]. In our study, the average seroprevalence of 25.8% (BW) and 32.4% (NW) was higher than the average RNA detection rate (24.1% and 20.5%, respectively). This discrepancy between seroprevalence and PUUV RNA detection can only partially be explained by the presence of maternal antibodies in juvenile individuals, as there were adult animals with anti-PUUV antibodies but without PUUV RNA. Therefore, we speculate that clearance of the PUUV infection had occurred in adult animals. Alternatively, an oscillation of viral RNA load might influence the RNA detection, as discussed earlier [24]. 

Phylogenetic and isolation-by-distance analysis of the novel PUUV S segment sequences confirmed strict spatial clustering. This phylogenetic clustering was already described in studies before [18,19,20,25,26]. In our study, the novel sequences from NW clustered with other sequences from NW in a single clade and the sequences from BW clustered in two clades: the major one with additional sequences from BW and a single sequence from the geographically distant western region of BW with sequences from France. Sequence type analysis indicated the temporal persistence of a single major sequence type over time, but the occurrence of additional minor types during outbreak years. These spatial and temporal patterns are in line with patterns of PUUV sequence variation in another endemic region close to Osnabrück [20]. 

Interestingly, sequence divergence varied within the S segment and the encoded N and NSs proteins. As expected, the overlapping NSs/N coding region seemed to result in a higher sequence conservation of the N protein when compared to the more downstream N-alone coding region [27]. Nevertheless, the position and length of the NSs ORF and the occurrence of multiple translation initiation codons was conserved despite a high level of nucleotide sequence divergence observed for PUUV strains of different European clades. Obviously, local evolution of PUUV strains was not only indicated in the non-overlapping region of the S segment, but also in the NSs/N overlapping region, as evidenced by SimPlot analysis. The spatial distribution of nucleotide sequence types may also indicate an “exchange” of the local PUUV strains between individuals at different plots. This was not only seen at forest plots, but also by the rare detection of PUUV-positive bank voles at grassland plots in BW. Here, bank voles from the same plot in NW showed different NSs nucleotide and amino acid sequence types, whereas in BW, voles from one plot carried mainly one NSs sequence type, indicating higher exchange or contact of territories in BW. This can be explained by the location of trapping plots in this study, as they were only a few hundred meters apart in BW. In NW, forest sites were not connected among each other and were more separated than in BW. However, we cannot exclude the influence of different population sizes or their fluctuations and of migration processes on these observations. Thus, larger populations of bank voles may contribute to a higher sequence variability, that is driven by the high mutation rate of RNA viruses, or re-populating of areas after bottleneck events can also increase variability [20]. Spontaneously occurring sequence types of NSs and N, only present in years with high vole abundance, were probably detected due to larger sample size in outbreak years and were not necessarily absent in non-outbreak years. In a previous study, land cover type influenced dispersal dynamics of PUUV, with forests facilitating and croplands impeding virus spread [28].

Usually, a hantavirus is found in a single reservoir species, although there is increasing evidence that some vole-associated orthohantaviruses might be capable of replication in several closely related rodent species. TULV was initially found in the common vole and its sibling species, the East European vole (*M. levis*) [29,30], but was later also detected in the field vole, the European pine vole (*M. subterraneus*) and even the European water vole (*Arvicola amphibius*) [31,32,33]. Molecular evidence for PUUV spillover infection was obtained in our study for a single field vole collected during the outbreak year 2010 at a forest plot in BW. None of the common voles were PUUV RNA-positive, and none of the bank voles were positive for TULV RNA, although in another study, TULV RNA was detected in several common and field voles (Schmidt and Reil et al., in preparation). These results confirm the host specificity of hantaviruses in general (for review, see Reference [10]), and of PUUV for the bank vole and TULV for common vole and related *Microtus* species in particular, which is consistent with the results of a recent in vitro study [34]. The very low proportion of PUUV spillover infection observed in our study (1 of 649) is in line with an experimental rodent infection study [35]. Furthermore, PUUV-reactive antibodies were not found here in yellow-necked mice and wood mice, although previous studies have detected anti-PUUV antibody positive yellow-necked mice occurring sympatrically with PUUV-infected bank voles in PUUV endemic regions in NW (city of Cologne) and Bavarian forest [22,36].

Viruses co-evolve with their hosts and the principle of overlapping genes has resulted in a highly efficient viral gene expression strategy and genome size minimization. Mechanisms such as leaky scanning (in the case of NSs) or usage of different reading frames have led to highly economical coding strategies [37]. The NSs protein is highly dependent on the N protein in terms of transcription as it uses the same mRNA by leaky scanning [38]. Our data show that the N protein of local PUUV strains is highly conserved. In particular, the residues 175–215 of the N protein are highly conserved, most likely due to their function in RNA binding [39,40]. The N-terminal part of N has interaction domains for RdRP [41] and Gc [42] but is also highly immunogenic. Therefore, the sequence evolution of the encoding region, that overlaps that of the NSs protein, needs to be well balanced for overall N and NSs functions. The high variability at amino acid residues 220–240 has also been observed in previous studies on N protein of TULV [2,43,44].

## 4. Materials and Methods 

### 4.1. Study Animals

Rodents were captured using snap traps in spring, summer and autumn 2010–2014 in BW, except for spring 2014, and in spring, summer and autumn 2010–2012 in NW, except for autumn 2012 (Figure 1a). In the two regions, three forest (F) and three grassland (G) plots were established (Figure 1b,c). A total of 851 bank voles, 397 yellow-necked mice, 68 wood mice, 176 common voles and 8 field voles were trapped in these two regions (Supplementary Table S2). From these animals, 36 bank voles were found dead during live trapping in BW nearby snap trapping plots (BWF2, BWF3) and were included in the study. Additionally, 32 bank voles were obtained during the outbreak years 2007 and 2012 from 10 plots in BW [16,17] (Figure 1a; Supplementary Table S1). All animals were dissected, and tissue and chest cavity lavage samples were collected according to standard protocols. Animals of ≤15 g were considered juvenile [14].

### 4.2. Serology

Investigation of chest cavity lavage samples from bank voles, yellow-necked mice and wood mice was done by IgG ELISA using recombinant N protein of PUUV strain BaWa, as described earlier [45]. The monoclonal antibody 5E11 was used as a positive control [46]. Sera of PUUV RT-PCR and IgG ELISA-negative bank vole and yellow-necked mouse were used as negative controls for serological investigation of bank voles and *Apodemus* mice, respectively. The definition of positive, negative and equivocal followed a previously introduced workflow [45].

### 4.3. Detection of Hantavirus RNA

For detection of PUUV nucleic acids, RNA was extracted from homogenized lung tissue using QIAzol Lysis Reagent (QIAGEN, Hilden, Germany) followed by S segment-specific RT-PCR that allows detection of RNA of PUUV, TULV and related viruses. Primers 342F (5’-TATGGTAATGTCCTTGATGT-3’) and 1102R (5’-GCCATDATDGTRTTYCTCAT-3’) were applied to amplify the main part of the N protein-coding region [17]. The amplification with Superscript TM III RT/Platinum Taq Mix (Invitrogen, Karlsruhe, Germany) followed the instructions of the manufacturer. Following reverse transcription at 50 °C for 30 min and denaturation at 94 °C for 2 min, cDNA was amplified in 40 cycles for 30 s at 94 °C, 30 s at 46 °C, 1 min at 68 °C, with a final extension for 10 min at 68 °C. For identification of PUUV, sequences were generated from each RT-PCR-positive animal (see Section 4.4). Few partial sequences of 504 nucleotides from BW and NW have been reported before ([18], accession numbers JN696363, JN696359, JN696362, JN696360, JN696370). For PUUV RNA-positive animals, the NSs-overlapping region was additionally amplified using the primers 40F (5’-CTGGAATGAGTGACTTAAC-3’) and 393R (5’-CTCCAATTGTATACCAATCT-3’) with the same cycler protocol.

### 4.4. Sequence, Phylogenetic and Statistical Analysis

Amplified PUUV cDNA was sequenced with the primers used for RT-PCR in four independent runs using the BigDye™ Terminator v1.1 Cycle Sequencing Kit (Thermo Fisher, Waltham, MA, USA) and the following reaction profile: 96 °C for 1 min, followed by 30 cycles of 96 °C for 15 s, 50 °C for 15 s and 60 °C for 90 s. Sequences (n = 226, accession numbers: MT453485-MT453710) were assembled and aligned using MEGA 10 [47]. Published sequences of other hantaviruses were obtained from GenBank. Phylogenetic trees were reconstructed with 213 non-identical partial S segment sequences of 465 nucleotides length (nucleotides 436–900 of the S segment, numbering according PUUV strain Sotkamo, accession number HE801633.1). A total of 39 unique sequences from the newly generated 226 individual sequences were used for phylogenetic reconstruction. Analysis was performed by Bayesian algorithms via MrBayes v.3.2.7 on the CIPRES online portal [21]. A mixed nucleotide substitution matrix was specified in four independent runs of 10^7^ generations. Phylogenetic relationships are shown as a maximum clade credibility phylogenetic tree with posterior probabilities for major nodes.

We investigated the extent of local transmission and evolution of PUUV by testing for isolation-by-distance patterns [19] within and among the sampling regions in BW and NW. Pairwise genetic distances were estimated based on the same above-mentioned 465 nucleotides-long S segment sequence in MEGA 7 [48], including transitions and transversions and assuming uniform mutation rates among sites. Geographic distances between sampling locations were calculated using the Geographic Distance Matrix Generator v. 1.2.3 [49]. We tested for statistical significance of the association between the half-matrices of both distance types with a Mantel test using R software [50]. The same software was used to plot the genetic distances against the geographic distances for each sequence pair.

For SimPlot analysis, NSs and N nucleotide and amino acid sequences from our study were compared among each other and with NSs and N sequences of 78 sequences from all PUUV clades obtained from GenBank National Center for Biotechnology Information NCBI (accession numbers: AB010730, AB010731, AB297665, AB433843, AB433845, AB675453, AB675463, AF063892, AF294652, AF367071, AF442613, AJ223369, AJ223371, AJ223374, AJ223375, AJ223380, AJ238790, AJ238791, AJ277030, AJ277031, AJ277033, AJ314598, AJ314599, AJ888751, AM695638, AY526219, AY954722, DQ016432, EU439968, FN377821, GQ339474, GQ339476, GQ339477, GQ339478, GQ339479, GQ339480, GQ339481, GQ339482, GQ339483, GQ339484, GQ339485, GQ339486, GQ339487, GU808824, GU808825, JN657228, JN657229, JN657230, JN657231, JN696358, JN696372, JN696373, JN696374, JN696375, JN831943, JQ319162, JQ319163, JQ319168, JQ319170, JQ319171, KJ994776, KT247592, KT247593, KT247595, KT247596, KT247597, L08804, M32750, U14137, U22423, Z21497, Z30702, Z30704, Z30705, Z46942, Z48586, Z69991, Z84204). For SimPlot analysis, a window size of 9 and a step size of 3 for nucleotide sequence analysis as well as a window size of 3 and step size of 1 for amino acid sequence analysis were used. Scripts for SimPlot analysis were written in R software [50].

Comparison of N and NSs sequences was done by grouping all PUUV sequences into sequence types. A sequence type is defined as a unique sequence found in a bank vole (or field vole) with at least one nucleotide/amino acid difference to any other sequence (Supplementary Tables S3 and S4). For statistical analysis of PUUV RNA detection and serological investigations, CI based on a binomial distribution were calculated. 

### 4.5. Ethical Statement 

Collection of samples was done according to relevant legislation and by permission of the federal authorities (permits: Regierungspräsidium Stuttgart 35–9185.82/0261; Landesamt für Natur, Umwelt und Verbraucherschutz Nordrhein-Westfalen 8.87–51.05.20.09.210).

## 5. Conclusions

Our study added novel knowledge on the fluctuation of PUUV prevalence in local bank vole populations during outbreak and non-outbreak years and the differences of sequence conservation and variability in the S segment regions encoding NSs and N, or N protein alone. The persistence of sequence types over time and the emergence of novel sequence types might be explained by bottleneck event-driven genetic drift or selection processes in the bank vole population that shape the genetic diversity of PUUV. These evolutionary processes need to be evaluated in future studies. The general conservation of the NSs ORF is in contrast to the high amino acid sequence variability, which raises questions about its functional consequences. There was a low frequency of spillover infections in general, but the PUUV spillover to one of eight field voles observed here indicated the necessity of further studies on the virus–host interaction on the cellular and organismic levels. Finally, our study increased our sequence knowledge for future identification of the geographical origin of human infections in high endemic regions of NW and BW [18,24].

## Supplementary Materials

The following are available online at https://www.mdpi.com/2076-0817/9/7/548/s1. Table S1: Trapping site and season information for PUUV-positive bank voles from Baden-Wuerttemberg from outbreak years 2007 and 2012. Table S2: Rodents trapped for PUUV investigations over a five-year period and results of serological tests (ELISA) and RNA detection (RT-PCR). Table S3: Assignment of nucleotide sequences to sequence types for PUUV strains from Baden-Wuerttemberg. Table S4: Assignment of nucleotide sequences to sequence types for PUUV strains from North Rhine-Westphalia. Figure S1: Amino acid sequence alignment of unique putative NSs proteins of Puumala orthohantavirus (PUUV) strains from Baden-Wuerttemberg (BW) and North Rhine-Westphalia (NW), and previously described PUUV isolates from Osnabrück region, Lower Saxony. Figure S2: Amino acid sequence alignment of unique partial N protein segments encoded by overlapping (a) or non-overlapping NSs ORF (b) parts of Puumala orthohantavirus (PUUV) strains from Baden-Wuerttemberg (BW) and North Rhine-Westphalia (NW), and previously described PUUV isolates from Osnabrück region, Lower Saxony.
