# Supplementary material for: Spatial and Temporal Evolutionary Patterns in Puumala Orthohantavirus (PUUV) S Segment
Source: Pathogens. 2020 Jul 8;9(7):548. doi: 10.3390/pathogens9070548 (PMC7400055; doi:10.3390/pathogens9070548)
Supplement: Supplementary file 1 [file pathogens-09-00548-s001.pdf]

pathogens

Spatial and Temporal Evolutionary Patterns in Puumala Orthohantavirus (PUUV) S segment

Table S1. Trapping site and season information for PUUV-positive bank voles from Baden-Wuerttemberg from outbreak years 2007 and 2012.

Animal Number	Trapping Site (Figure 1a)	Trapping Season	Reference
Mu07/458	4 Zußdorf-Wilhelmsdorf	2007 summer	[1]
Mu07/459	4 Zußdorf-Wilhelmsdorf	2007 summer	[1]
Mu07/460	4 Zußdorf-Wilhelmsdorf	2007 summer	[1]
Mu07/473	4 Zußdorf-Wilhelmsdorf	2007 summer	[1]
Mu07/476	4 Zußdorf-Wilhelmsdorf	2007 summer	[1]
Mu07/477	4 Zußdorf-Wilhelmsdorf	2007 summer	[1]
Mu07/492	4 Zußdorf-Wilhelmsdorf	2007 summer	[1]
Mu07/503	6 Michelbach	2007 summer	[1]
Mu07/533	10 Steinheim B	2007 summer	[1]
Mu07/535	10 Steinheim B	2007 summer	[1]
Mu07/538	10 Steinheim B	2007 summer	[1]
Mu07/546	10 Steinheim B	2007 summer	[1]
KS12/2547	9 Steinheim A	2012 summer	[2]
KS12/2573	9 Steinheim A	2012 summer	[2]
KS12/2574	9 Steinheim A	2012 summer	[2]
KS12/2585	7 Geislingen-Stoetten	2012 summer	[2]
KS12/2587	3 Stuttgart-Buesnau	2012 summer	[2]
KS12/2592	2 Moessingen-Belsen	2012 summer	[2]
KS12/2594	2 Moessingen-Belsen	2012 summer	[2]
KS12/2595	7 Geislingen-Stoetten	2012 summer	[2]
KS12/2606	3 Stuttgart-Buesnau	2012 summer	[2]
KS12/2608	3 Stuttgart-Buesnau	2012 summer	[2]
KS12/2709	2 Moessingen-Belsen	2012 summer	[2]
KS13/536	1 Kenzingen	2012 summer	[2]
KS13/651	1 Kenzingen	2012 summer	[2]
KS13/656	1 Kenzingen	2012 summer	[2]
KS13/692	8 Crailsheim-Rossfeld	2012 summer	[2]
KS13/695	8 Crailsheim-Rossfeld	2012 summer	[2]
KS13/700	8 Crailsheim-Rossfeld	2012 summer	[2]
KS13/703	5 Goeppingen	2012 autumn	[2]
KS13/704	5 Goeppingen	2012 autumn	[2]
KS13/711	5 Goeppingen	2012 autumn	[2]

PUUV S segment sequences were generated for all 32 animals.

Table S2. Rodents trapped for PUUV investigations over a five-year period and results of serological tests (ELISA) and RNA detection (RT-PCR).

			PUUV-IgG ELISA		RT-PCR	
**Trapping Region**	**Species**	**Year**	**Number of Positive/total Number Investigated Animals**	**Seroprevalence (%) and (95% CI)**	**Number of Positive/total Number Investigated Animals**	**RNA prevalence (%) and (95% CI)**
Baden-Wuerttemberg (BW)	Bank vole (*Clethrionomys glareolus*)	2010	77/266	29.0 (23.8–34.7)	74/266	27.8 (22.8–33.5)
		2011	2/32	5.4 (1.7–20.2)	2/32	6.3 (1.7–20.2)
		2012	45/163	27.6 (21.3–34.9)	44/163	27.0 (20.8–34.3)
		2013	0/23	0 (0–14.3)	0/23	0 (0–14.3)
		2014	9/31	29.0 (16.1–46.6)	4/31	12.9 (5.13–28.9)
		total	133/515	25.8 (22.2–29.8)	124/515	24.1 (20.6–28.0)
	Yellow-necked mouse (*Apodemus flavicollis*)	2010	0/88	0 (0–4.2)	n.d.	
		2011	0/24	0 (0–13.9)	n.d.	
		2012	0/103	0 (0–3.6)	n.d.	
		2013	0/20	0 (0–16.1)	n.d.	
		2014	0/20	0 (0–16.1)	n.d.	
		total	0/255	0 (0–1.5)		
	Wood mouse (*Apodemus sylvaticus*)	2010	0/4	0 (0–49.0)	n.d.	
		2011	0/10	0 (0–27.8)	n.d.	
		2012	0/3	0 (0–56.2)	n.d.	
		2013	0/5	0 (0–43.5)	n.d.	
		2014	0/1	0 (0–79.4)	n.d.	
		total	0/23	0 (0–14.3)		
	Common vole (*Microtus arvalis*)	2010	n.d.		0/45	0 (0–7.9)
		2011	n.d.		0/78	0 (0–4.7)
		2012	n.d.		0/8	0 (0–32.4)
		2013	not trapped		not trapped	
		2014	n.d.		0/27	0 (0–12.5)
		total			0/158	0 (0–2.4)
	Field vole (*Microtus agrestis*)	2010	n.d.		1/6	16.67 (3.0-56.4)
		2011	n.d.		0/1	0 (0–79.4)
		2012	not trapped		not trapped	
		2013	not trapped		not trapped	
		2014	not trapped		not trapped	
		total			1/7	14.3 (2.6–51.3)

North Rhine-Westphalia (NW)	Bank vole (*Clethrionomys glareolus*)	2010	86/249	34.5 (28.9–40.6)	55/249	22.1 (17.4–27.6)
		2011	2/32	6.3 (1.7–20.2)	1/32	3.1 (0.6–15.7)
		2012	21/55	38.2 (26.5–51.4)	13/55	23.6 (14.4–36.4)
		total	109/336	32.4 (27.7–37.6)	69/336	20.5 (16.6–25.2)
	Yellow-necked mouse (*Apodemus flavicollis*)	2010	0/96	0 (0–3.9)	0/96	0 (0–3.9)
		2011	0/9	0 (0–29.9)	0/9	0 (0–29.9)
		2012	0/37	0 (0–9.4)	0/37	0 (0–9.4)
		total	0/142	0 (0–2.6)	0/142	0 (0–2.6)
	Wood mouse (*Apodemus sylvaticus*)	2010	0/24	0 (0–13.8)	0/24	0 (0–13.8)
		2011	0/11	0 (0–25.9)	0/11	0 (0–25.9)
		2012	0/10	0 (0–27.8)	0/10	0 (0–27.8)
		total	0/45	0 (0–7.9)	0/45	0 (0–7.9)
	Common vole (*Microtus arvalis*)	2010	n.d.		0/9	0 (0–29.9)
		2011	n.d.		0/6	0 (0–39.0)
		2012	n.d.		0/3	0 (0–56.2)
		total			0/18	0 (0–17.6)
	Field vole (*Microtus agrestis*)	2010	n.d.		not trapped	
		2011	n.d.		0/1	0 (0–79.4)
		2012	n.d.		not trapped	
		total			0/1	0 (0–79.4)

n.d., not done; CI, confidence interval; ELISA, enzyme-linked immunosorbent assay; RT-PCR, reverse transcription-polymerase chain reaction. In common voles and field voles, TULV was found by sequencing and results will be included in a separate publication (Schmidt and Reil et al., in prep.). PUUV S segment sequences were generated for all 194 RNA-positive animals, including 193 bank voles and a field vole.

Table S3. Assignment of nucleotide sequences to sequence types for PUUV strains from Baden-Wuerttemberg.

Sequence types NSs	Sequence types N/NSs	Sequence types N
**NSs-nt-BW1:** KS10/972, KS10/978, KS10/979, KS10/983, KS10/984, KS10/987, KS10/999, KS10/1001, KS10/1002, KS10/1006, KS10/1008, KS10/1011, KS10/1015, KS10/1017, KS10/1025, KS10/1026, KS10/1027, KS10/1029, KS10/1035, KS10/1039, KS10/1048, KS10/1049, KS10/1053, KS10/1054, KS10/1056, KS10/1060, KS10/1064, KS10/1065, KS10/1066, KS10/1076, KS10/1077, KS10/1078, KS10/1081, KS10/1082, KS10/1084, KS10/1086, KS10/1089, KS10/1093, KS10/1098, KS10/1099, KS10/1103, KS10/1109, KS10/1114, KS10/1115, KS10/1118, KS10/1121, KS10/1124, KS10/1127, KS10/1128, KS10/1135, KS10/1850, KS10/1854, KS10/1865, KS10/1869, KS10/1871, KS10/1876, KS10/1886, KS10/1887, KS10/1919, KS10/1928, KS10/1935, KS10/3423, KS10/3440, KS10/3556, KS10/3562, KS10/3571, KS11/2037, KS12/1679, KS12/1680, KS12/1681, KS12/1682, KS12/1683, KS12/1685, KS12/1686, KS12/1687, KS12/1688, KS12/1689, KS12/1696, KS12/1700, KS12/1702, KS12/1703, KS12/1705, KS12/1706, KS12/1709, KS12/1713, KS12/1716, KS12/1737, KS12/1740, KS12/1741, KS12/1742, KS12/1745, KS12/1749, KS12/1753, KS12/1756, KS12/1757, KS12/1768, KS12/1769, KS12/1774, KS12/2326, KS12/2327, KS12/2331, KS12/2414, KS12/2427, KS12/2429, KS12/2443, KS13/258, KS13/267, KS13/281, KS13/289, KS15/207, KS15/315, KS15/317, KS10/1083, KS11/2364	**N/NSs-nt-BW1:** KS10/972, KS10/978, KS10/979, KS10/983, KS10/984, KS10/987, KS10/999, KS10/1001, KS10/1002, KS10/1006, KS10/1008, KS10/1011, KS10/1015, KS10/1017, KS10/1025, KS10/1026, KS10/1027, KS10/1029, KS10/1035, KS10/1039, KS10/1048, KS10/1049, KS10/1053, KS10/1054, KS10/1056, KS10/1060, KS10/1064, KS10/1065, KS10/1066, KS10/1076, KS10/1077, KS10/1078, KS10/1081, KS10/1082, KS10/1084, KS10/1086, KS10/1089, KS10/1093, KS10/1098, KS10/1099, KS10/1103, KS10/1109, KS10/1114, KS10/1115, KS10/1118, KS10/1121, KS10/1124, KS10/1127, KS10/1128, KS10/1135, KS10/1850, KS10/1854, KS10/1865, KS10/1869, KS10/1871, KS10/1876, KS10/1886, KS10/1887, KS10/1919, KS10/1928, KS10/1935, KS10/3423, KS10/3440, KS10/3556, KS10/3562, KS10/3571, KS11/2037, KS12/1679, KS12/1680, KS12/1681, KS12/1682, KS12/1683, KS12/1685, KS12/1686, KS12/1687, KS12/1688, KS12/1689, KS12/1696, KS12/1700, KS12/1702, KS12/1703, KS12/1705, KS12/1706, KS12/1709, KS12/1713, KS12/1716, KS12/1737, KS12/1740, KS12/1741, KS12/1742, KS12/1745, KS12/1749, KS12/1753, KS12/1756, KS12/1757, KS12/1768, KS12/1769, KS12/1774, KS12/2326, KS12/2327, KS12/2331, KS12/2414, KS12/2427, KS12/2429, KS12/2443, KS13/258, KS13/267, KS13/281, KS13/289, KS15/207, KS15/315, KS15/317, KS10/1083, KS11/2364	**N-nt-BW1:** KS10/978, KS10/979, KS10/984, KS10/987, KS10/1001, KS10/1002, KS10/1006, KS10/1015, KS10/1017, KS10/1026, KS10/1029, KS10/1035, KS10/1048, KS10/1049, KS10/1053, KS10/1054, KS10/1056, KS10/1076, KS10/1077, KS10/1081, KS10/1082, KS10/1084, KS10/1093, KS10/1098, KS10/1099, KS10/1103, KS10/1109, KS10/1114, KS10/1115, KS10/1124, KS10/1127, KS10/1128, KS10/1135, KS10/1854, KS10/1865, KS10/1871, KS10/1886, KS10/3440, KS10/3556, KS10/3562, KS10/3571, KS12/1679, KS12/1680, KS12/1681, KS12/1682, KS12/1683, KS12/1685, KS12/1686, KS12/1687, KS12/1688, KS12/1689, KS12/1696, KS12/1702, KS12/1703, KS12/1705, KS12/1706, KS12/1709, KS12/1713, KS12/1716, KS12/1737, KS12/1740, KS12/1741, KS12/1742, KS12/1749, KS12/1753, KS12/1768, KS12/1769, KS12/2326, KS12/2327, KS12/2331, KS12/2383, KS12/2414, KS12/2427, KS12/2429, KS12/2443, KS13/233, KS13/267, KS13/281, KS15/207, KS15/307, KS15/315, KS15/317
**NSs-nt-BW2-T20G:** KS10/994, KS10/1019	**N/NSs-nt-BW2-T20G:** KS10/994, KS10/1019	**N-nt-BW2:** KS10/972, KS10/994, KS10/1019, KS10/1935
**NSs-nt-BW3-T218A:** KS10/1068, KS10/1100, KS10/1848, KS10/1851, KS10/3428	**N/NSs-nt-BW3-T218A:** KS10/1068, KS10/1100, KS10/1848, KS10/1851, KS10/3428	**N-nt-BW3:** KS10/983, KS10/1011, KS10/1025, KS10/1027, KS10/1039, KS10/1118
**NSs-nt-BW4-C221T:** KS12/2383, KS13/233	**N/NSs-nt-BW4-C221T:** KS12/2383, KS13/233	**N-nt-BW4:** KS10/999
**NSs-nt-BW5-G179A:** KS15/307	**N/NSs-nt-BW5-G179A:** KS15/307	**N-nt-BW5:** KS10/1008, KS10/1060, KS10/1065, KS10/1086, KS10/1089, KS10/1121, KS10/1850, KS10/1876, KS10/1887, KS10/3423
		**N-nt-BW6:** KS10/1064
		**N-nt-BW7:** KS10/1066, KS11/2037, KS11/2364, KS13/258
		**N-nt-BW8:** KS10/1068, KS10/1100, KS10/1848, KS10/1851, KS10/3428
		**N-nt-BW9:** KS10/1869
		**N-nt-BW10:** KS10/1083
		**N-nt-BW11:** KS10/1078, KS12/1700, KS12/1757, KS12/1774
		**N-nt-BW12:** KS10/1919, KS10/1928

**N-nt-BW13:** KS12/1745, KS12/1756
**N-nt-BW14:** VKS13/289

Animal numbers of identical partial S segment nucleotide sequences are grouped together as one sequence type. Colors of text shade indicate the sequence type shown in Figure 5a. Light gray background indicates the cumulative amino acid sequence type N/NSs-aa-BW1 or N-aa-BW1. The amino acid sequences of the NSs-aa sequence types are given in Supplementary Figure S1, the amino acid sequences of the N-aa sequence types (overlapping and non-overlapping NSs) are shown in Supplementary Figure S2a and b. Nucleotide exchanges in the N/NSs overlapping region in comparison to sequence type BW1 are given next to sequence type names (residue in sequence type BW1/position/residue in the other sequence type).

Table S4. Assignment of nucleotide sequences to sequence types for PUUV strains from North Rhine-Westphalia

Sequence types NSs	Sequence types N/NSs	Sequence types N
**NSs-nt-NW1:** KS10/2030, KS10/2036, KS10/2038, KS10/2040, KS10/2059, KS10/2060, KS10/2061, KS10/2067, KS10/2073, KS10/2083, KS10/2092, KS10/2094, KS10/2095, KS10/2098, KS10/2099, KS10/2100, KS10/2103, KS10/2104, KS10/2159, KS10 2167, KS10/2169, KS10/2171, KS10/2173, KS10/2175, KS10/2178, KS10/2179, KS10/2186, KS10/2188, KS10/2195, KS10/2199, KS10/2200, KS10/2203, KS10/2204, KS10/2219, KS10/2222, KS10/2228, KS10/2234, KS10/2235, KS10/2243, KS10/2668, KS10/2705, KS10/2990, KS11/2251, KS12/1808, KS12/2478, KS10/2170	**N/NSs-nt-NW1:** KS10/2030, KS10/2036, KS10/2038, KS10/2040, KS10/2059, KS10/2060, KS10/2061, KS10/2067, KS10/2073, KS10/2083, KS10/2092, KS10/2094, KS10/2095, KS10/2098, KS10/2099, KS10/2100, KS10/2103, KS10/2104, KS10/2159, KS10 2167, KS10/2169, KS10/2171, KS10/2173, KS10/2175, KS10/2178, KS10/2179, KS10/2186, KS10/2188, KS10/2195, KS10/2199, KS10/2200, KS10/2203, KS10/2204, KS10/2219, KS10/2222, KS10/2228, KS10/2234, KS10/2235, KS10/2243, KS10/2668, KS10/2705, KS10/2990, KS11/2251, KS12/1808, KS12/2478, KS10/2170	**N-nt-NW1:** KS10/2001
**NSs-nt-NW2-A44G:** KS10/2001, KS10/2010, KS10/2020, KS10/2022, KS10/2025, KS10/2028, KS10/2090, KS10/2102, KS10/2174, KS10/2190, KS10/2192, KS10/2592, KS12/1777, KS12/1816, KS12/2471, KS12/2476, KS12/2515, KS12/2517, KS12/2520, KS12/2522	**N/NSs-nt-NW2-A44G:** KS10/2001, KS10/2010, KS10/2020, KS10/2022, KS10/2025, KS10/2028, KS10/2090, KS10/2102, KS10/2174, KS10/2190, KS10/2192, KS10/2592, KS12/1777, KS12/1816, KS12/2471, KS12/2476, KS12/2515, KS12/2517, KS12/2520, KS12/2522	**N-nt-NW2:** KS10/2228, KS10/2235
**NSs-nt-NW3-G59A:** KS12/1778, KS12/2510	**N/NSs-nt-NW3-G59A:** KS12/1778, KS12/2510	**N-nt-NW3:** KS10/2020, KS10/2022, KS10/2025, KS10/2028, KS10/2090, KS10/2102, KS10/2190, KS10/2192
**NSs-nt-NW4-A85G:** KS12/2519	**N/NSs-nt-NW4-A85G:** KS12/2519	**N-nt-NW4:** KS10/2030, KS10/2038, KS10/2159, KS10/2175, KS10/2186, KS10/2188, KS10/2199, KS10/2203, KS10/2204, KS10/2243, KS10/2705, KS11/2251, KS12/1808, KS12/2478
		**N-nt-NW5:** KS10/2036, KS10/2040
		**N-nt-NW6:** KS10/2059, KS10/2060, KS10/2061, KS10/2073, KS10/2083, KS10/2092, KS10/2094, KS10/2095, KS10/2098, KS10/2099, KS10/2100, KS10/2103, KS10/2104, KS10/2167, KS10/2169, KS10/2170, KS10/2171, KS10/2173, KS10/2178, KS10/2179, KS10/2195, KS10/2200, KS10/2219, KS10/2222, KS10/2234, KS10/2668, KS10/2990
		**N-nt-NW7:** KS10/2067
		**N-nt-NW8:** KS10/2174
		**N-nt-NW9:** KS10/2010, KS10/2592
		**N-nt-NW10:** KS12/1777
		**N-nt-NW11:** KS12/1778, KS12/2510
		**N-nt-NW12:** KS12/1816, KS12/2515, KS12/2517, KS12/2519, KS12/2520, KS12/2522
		**N-nt-NW13:** KS12/2471, KS12/2476

Animal numbers of identical partial S segment nucleotide sequences are grouped together as one sequence type. Colors of text shade indicate the sequence type shown in Figure 5b. Light gray background indicates the cumulative major amino acid sequence types N/NSs-aa-NW1 and N-aa-NW1. Dark grey background shows the cumulative minor amino acid sequence type N-aa-NW9/11/13. The amino acid sequences of the NSs-aa sequence types are given in Supplementary

Figure S1, the amino acid sequences of the N-aa sequence types (overlapping and non-overlapping NSs) are shown in Supplementary Figure S2a and b. Nucleotide exchanges in the N/NSs overlapping region in comparison to sequence type NW1 are given next to sequence type names (residue in sequence type NW1/position/residue in the other sequence type).

Figure S1. Amino acid sequence alignment of unique putative NSs proteins of Puumala orthohantavirus (PUUV) strains from Baden-Wuerttemberg (BW) and North Rhine-Westphalia (NW) and previously described PUUV isolates from Osnabrück region, Lower Saxony.

```
                                         10        20        30        40        50        60        70        80        90
                                 ....|....|....|....|....|....|....|....|....|....|....|....|....|....|....|....|....|....|.
NSs-aa-BW1                       MNNNLLLPDKSSRMQKRRWKWTQMTLTKTHYKQGNRQCQHWKINSQITSEEWQMLCPGKRWILNLLIRLGLNLTTILKSDQVLGMEMSLM*
NSs-aa-BW2                       ......W...................................................................................*
NSs-aa-BW3                       ........................................................................Q.................*
NSs-aa-BW4                       .........................................................................M................*
NSs-aa-BW5                       ...........................................................K..............................*
Mu07/458 Zußdorf-Wilhelmsdorf 4  .................Q.R.......................S..................................N...........*
Mu07/476 Zußdorf-Wilhelmsdorf 4  ...................R.......................S.....G............................N...........*
Mu07/503 Michelbach 6            ......WL..........................................K...........................N...........*
Mu07/535 Steinheim 10            ............................I.................................................N..AF.......*
KS12/2547 Steinheim 9            ............................I............................................M....N..AF.......*
KS12/2574 Steinheim 9            ............................I.................................................N..AF.......*
KS12/2587 Stuttgart-Buesnau 3    ............................I......K..........................................N..AS.......*
KS12/2592 Moessingen-Belsen 2    ...............R...........................................K...................N.P.........*
KS13/536 Kenzingen 1             ..........N.K......R........I.C...........T.L.TSR..........K......T......M..SRR..A.D......*
KS13/692 Crailsheim-Rossfeld 8   ......WL..........................................K........K...................N...........*
NSs-aa-NW1                       .... W....NL.  RR.Q.R..........C....K.......K..T............K. S.........MITSRN. AS.......*
NSs-aa-NW2                       .... W....NL...R.Q.R..........C....K.......K..T............K...S.........MITSRN. AS.......*
NSs-aa-NW3                       .... W....NL...R.Q............C....K.......K..T............K...S.........MITSRN. AS.......*
NSs-aa-NW4                       .... W....NL...R.Q.R........A.C....K.......K..T............K...S.........MITSRN. AS.......*
MN639739 PUUV Osnabrück/V29      .... W..G. L...R.Q.R.......RI.C.P..K..... T.L.T............K...S..........ITSRNG. S..G....*
MN639742 PUUV Osnabrück/M43      .... W..G. L...R.Q.R.......RI.C.P..K..... T.L.T............K...S..........ITSRNG. S.......*
```

The main amino acid sequence type of NSs served as a reference (NSs-aa-BW1). Identical amino acid residues are shown as dots. NSs sequences from Goeppingen, Geislingen and additional samples from other trapping sites were identical to the shown sequences from BW. *, stop codon; putative methionine start codons are framed in red.

Figure S2. Amino acid sequence alignment of unique partial N protein segments encoded by overlapping (a) or non-overlapping NSs ORF (b) parts of Puumala orthohantavirus (PUUV) strains from Baden-Wuerttemberg (BW) and North Rhine-Westphalia (NW) and previously described PUUV isolates from Osnabrück region, Lower Saxony.

a

```
                                        22        32        42        52        62        72        82        92       102
                                ....|....|....|....|....|....|....|....|....|....|....|....|....|....|....|....|....|....|.
N/NSs-aa-BW1                    HEQQLVVARQKLKDAEKAVEMDPDDVNKNTLQARQQTVSALEDKLADYKRRMADAVSRKKMDTKPTDPTGIEPDDHLKERSSLRYGNVLDV
KS13/536_Kenzingen 1            ...............................................F...........................................
KS13//692_Crailsheim-Rossfeld   ..................................................K........................................
Mu07/535_Steinheim 10           .................................................................A.........................
N/NSs-aa-NW1                    ............................................I..............................................
N/NSs-aa-NW4                    ............................S...............I..............................................
MN639739 PUUV Osnabrück/V29     ...........................................................................................
```

b

```
                                       113       123       133       143       153       163       173       183       193       203
                                ....|....|....|....|....|....|....|....|....|....|....|....|....|....|....|....|....|....|....|....|
N-aa-BW1                        AIDIEEPSGQTADWYTIGVYVIGFTLPIILKALYMLSTRGRQTVKENKGTRIRFKDDTSFEDINGIRRPKHLYVSMPTAQSTMKAEELTPGRFRTIVCGL
N-aa-BW6                        ....................................................................................................
N-aa-BW7                        ....................................................................................................
N-aa-BW8                        ....................................................................................................
N-aa-BW9                        ....................................................................................................
Mu07/458 Zußdorf-Wilhelmsdorf 4 .....................M..............................................................................
Mu07/476 Zußdorf-Wilhelmsdorf 4 ....................................................................................................
Mu07/533 Steinheim 10           .....................M..............................................................................
KS12/2547 Steinheim 9           ....................................................................................................
KS12/2574 Steinheim 9           ....................................................................................................
KS12/2585 Geislingen-Stoetten 7 ....................................................................................................
KS12/2587 Stuttgart-Buesnau 3   ....................................................................................................
KS12/2592 Moessingen-Belsen 2   ....................................................................................................
KS12/2594 Moessingen-Belsen 2   ....................................................................................................
KS13/536 Kenzingen 1            .........................I..........................................................................
KS13/692 Crailsheim-Rossfeld 8  ....................................................................................................
N-aa-NW1                        ....................................................................................................
N-aa-NW7                        ....................................................................................................
N-aa-NW9                        ....................................................................................................
MN639739 PUUV Osnabrück/V29     ....................................................................................................
MN639742 PUUV Osnabrück/M43     ....................................................................................................
```

```
                                      213       223       233       243       253       263       273       283       293       303
                               ....|....|....|....|....|....|....|....|....|....|....|....|....|....|....|....|....|....|....|....|
N-aa-BW1                       FPTQIQVRNIMSPVMGVIGFSFFVKDWTERIGEFMERDCPFIKPEIKPGTPAQEAEFLKRNKVYFMQRQDVLDKNHVADIDKLIDYAASGDPTSPDNIES
N-aa-BW6                       ......................................................................................T.............
N-aa-BW7                       ................................................................................................T...
N-aa-BW8                       .....................................E..............................................................
N-aa-BW9                       .....................................................................................H..............
Mu07/458 Zußdorf-Wilhelmsdorf 4 ...............................K...DKE..............................................................
Mu07/476 Zußdorf-Wilhelmsdorf 4 ...............................R....KE..............................................................
Mu07/533 Steinheim 10          ...........................A..VK....KE........R.....................................................
KS12/2547 Steinheim 9          ...........................A...K....KE........R.....................................................
KS12/2574 Steinheim 9          ...........................A...K....KE........R.....................................................
KS12/2585 Geislingen-Stoetten 7 ...........................A...R....KE........R.....................................................
KS12/2587 Stuttgart-Buesnau 3  ..............................VR....KE........R.....................................................
KS12/2592 Moessingen-Belsen 2  ...............................R....KE.......................R......................................
KS12/2594 Moessingen-Belsen 2  ...............................R....KE.......................R........I.............................
KS13/536 Kenzingen 1           ...........................AD..R....KE.......V................I.....................................
KS13/692 Crailsheim-Rossfeld 8 .............................. R.... E..............................................................
N-aa-NW1                       ...........................AD. R... KE.......V......................................................
N-aa-NW7                       ...........................AD. R....KE.......V...................................N..................
N-aa-NW9                       ...........................AD. R....KE.......V............R.........................................
MN639739 PUUV Osnabrück/V29    ...........................AD..R....KE.......V......................................................
MN639742 PUUV Osnabrück/M43    ...........................A...R....KE.......V......................................................

                                      313       323       332
                               ....|....|....|....|....|....|....|.
N-aa-BW1                       PNAPWVFACAPDRCPPTCIYVAGMAELGAFFSILQD
N-aa-BW6                       ....................................
N-aa-BW7                       ....................................
N-aa-BW8                       ....................................
N-aa-BW9                       ....................................
Mu07/458 Zußdorf-Wilhelmsdorf 4 ....................................
Mu07/476 Zußdorf-Wilhelmsdorf 4 ....................................
Mu07/533 Steinheim 10          ....................................
KS12/2547 Steinheim 9          ....................................
KS12/2574 Steinheim 9          ...........................D........
KS12/2585 Geislingen-Stoetten 7 ....................................
KS12/2587 Stuttgart-Buesnau 3  ....................................
KS12/2592 Moessingen-Belsen 2  ....................................
KS12/2594 Moessingen-Belsen 2  ....................................
KS13/536 Kenzingen 1           ....................................
KS13/692 Crailsheim-Rossfeld 8 ....................................
N-aa-NW1                       ....................................
N-aa-NW7                       ....................................
N-aa-NW9                       ....................................
MN639739 PUUV Osnabrück/V29    ....................................
MN639742 PUUV Osnabrück/M43    ....................................
```

The main amino acid sequence type of N from BW served as a reference (N-aa-BW1). Identical amino acid residues are shown as dots. N sequences from Goeppingen and Michelbach, and additional samples from other trapping sites were identical to the shown sequences from BW.

References

1. Drewes, S.; Ali, H.S.; Saxenhofer, M.; Rosenfeld, U.M.; Binder, F.; Cuypers, F.; Schlegel, M.; Rohrs, S.; Heckel, G.; Ulrich, R.G. Host-Associated Absence of Human Puumala Virus Infections in Northern and Eastern Germany. *Emerging Infect. Dis.* **2017**, *23,* 83–86, doi:10.3201/eid2301.160224.
2. Drewes, S.; Turni, H.; Rosenfeld, U.M.; Obiegala, A.; Strakova, P.; Imholt, C.; Glatthaar, E.; Dressel, K.; Pfeffer, M.; Jacob, J., et al. Reservoir-Driven Heterogeneous Distribution of Recorded Human Puumala virus Cases in South-West Germany. *Zoonoses. Public. Health.* **2017**, *64*, 381–390 doi:10.1111/zph.12319.

© 2020 by the author. Licensee MDPI, Basel, Switzerland. This article is an open access article distributed under the terms and conditions of the Creative Commons Attribution (CC BY) license (http://creativecommons.org/licenses/by/4.0/).
